# MEK Inhibition in a Pilocytic Astrocytoma With a Rare *KRAS Q61R* Mutation in a Young Adult Patient: A Case Report

**DOI:** 10.1200/PO.24.00174

**Published:** 2024-06-21

**Authors:** Katrina Roberto, Julia Keith, Adrian Levine, Farhad Pirouzmand, Hany Soliman, Mary Jane Lim-Fat

**Affiliations:** ^1^ Division of Neurology, Department of Medicine, Sunnybrook Health Sciences Centre, University of Toronto, Toronto, ON, Canada; ^2^ Department of Laboratory Medicine and Pathobiology, Sunnybrook Health Sciences Centre, University of Toronto, Toronto, ON, Canada; ^3^ Department of Pediatric Laboratory Medicine, The Hospital for Sick Children, Toronto, ON, Canada; ^4^ Division of Neurosurgery, Department of Surgery, Sunnybrook Health Sciences Centre, University of Toronto, Toronto, ON, Canada; ^5^ Department of Radiation Oncology, Sunnybrook Health Sciences Centre, University of Toronto, Toronto, ON, Canada

## Abstract

This case illustrates the utility and impact of molecular testing and molecular tumor board discussion in the management of AYA patients with brain tumors.

## Introduction

Pilocytic astrocytoma (PA) is a circumscribed astrocytic neoplasm classified as a WHO grade 1 tumor that develops predominantly in children.^1^ It accounts for 15.9% of all CNS tumors among children and adolescents age 0-19 years with a median age of diagnosis at 11 years. The average annual age-adjusted incidence rate of PA is 1.10 in children (0-14 years), 0.28 in the adolescent and young adult (AYA) group (15-39 years), and 0.08 in older adults (40+ years).^2^ Mitogen-activated protein kinase (MAPK) pathway alterations, such as *BRAF* fusions, are central to its oncogenesis and targeted therapy with BRAF and MEK inhibitors has been increasingly used after demonstration of their safety and efficacy in clinical trials.^3-5^ In this report, we describe a case of a young adult female patient with a PA identified to have an oncogenic *KRAS* mutation treated with up-front MEK inhibition.

## Case Presentation

**FIG 1.**
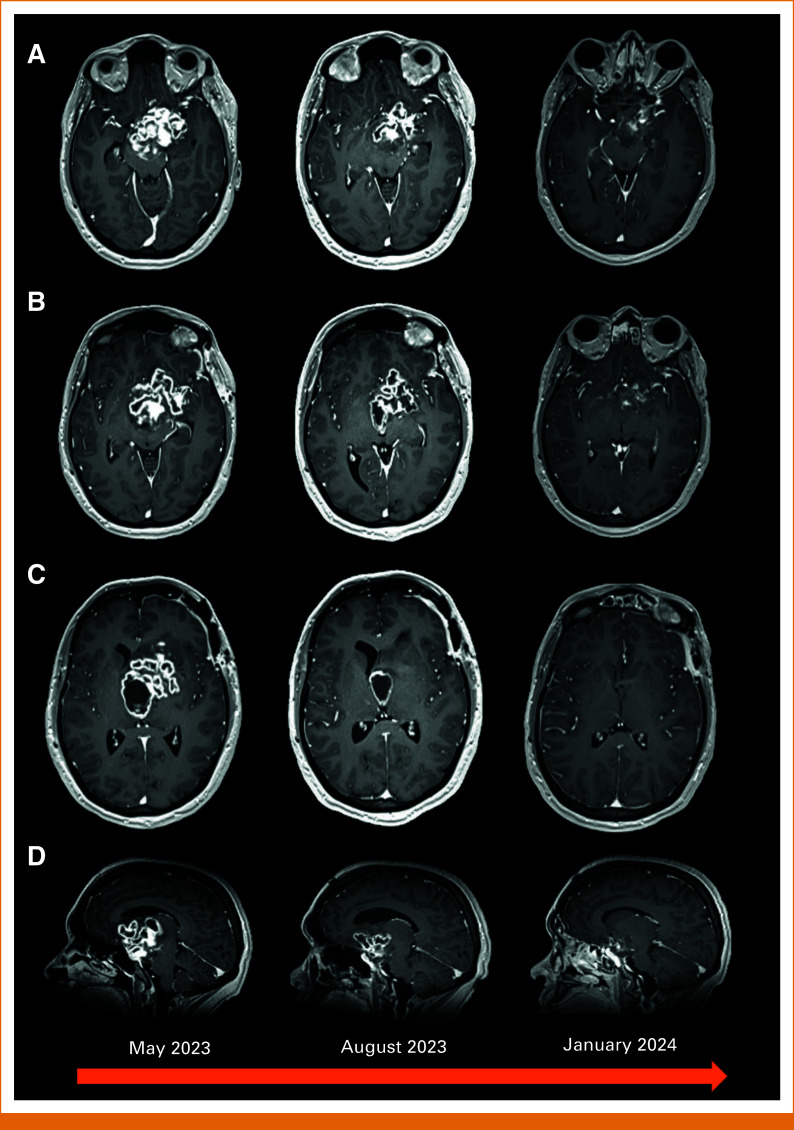
Magnetic resonance imaging of the brain: (A) Axial postcontrast at the level of the midbrain, (B) at the level of the hypothalamus, (C) at the level of the third ventricle, and (D) sagittal postcontrast. From left to right, the images show the treatment response as depicted in the significant reduction in size of the irregularly enhancing and multilobulated cystic suprasellar mass after 6 months of trametinib.

A 28-year-old female patient with no significant medical or family history presented to the emergency department with a 3-month history of progressive nonremitting headaches, right-sided visual field defect, and lethargy. Magnetic resonance imaging (MRI) of the brain revealed a large multiloculated multicystic enhancing lesion in the suprasellar region associated with third ventricular obstruction and hydrocephalus (Fig 1). She was urgently taken to the operating room for a subtotal resection of the tumor. She tolerated the procedure well and was discharged with improved neurological status. Two weeks postoperatively, however, she was readmitted because of a first-ever generalized tonic-clonic seizure. Repeat neuroimaging indicated progressive obstructive hydrocephalus from the tumor which showed further increase in size from baseline. She thereafter underwent right frontal ventriculostomy and septostomy with external ventricular drainage insertion that was eventually converted into a right frontal ventriculoperitoneal shunt.

**FIG 2.**
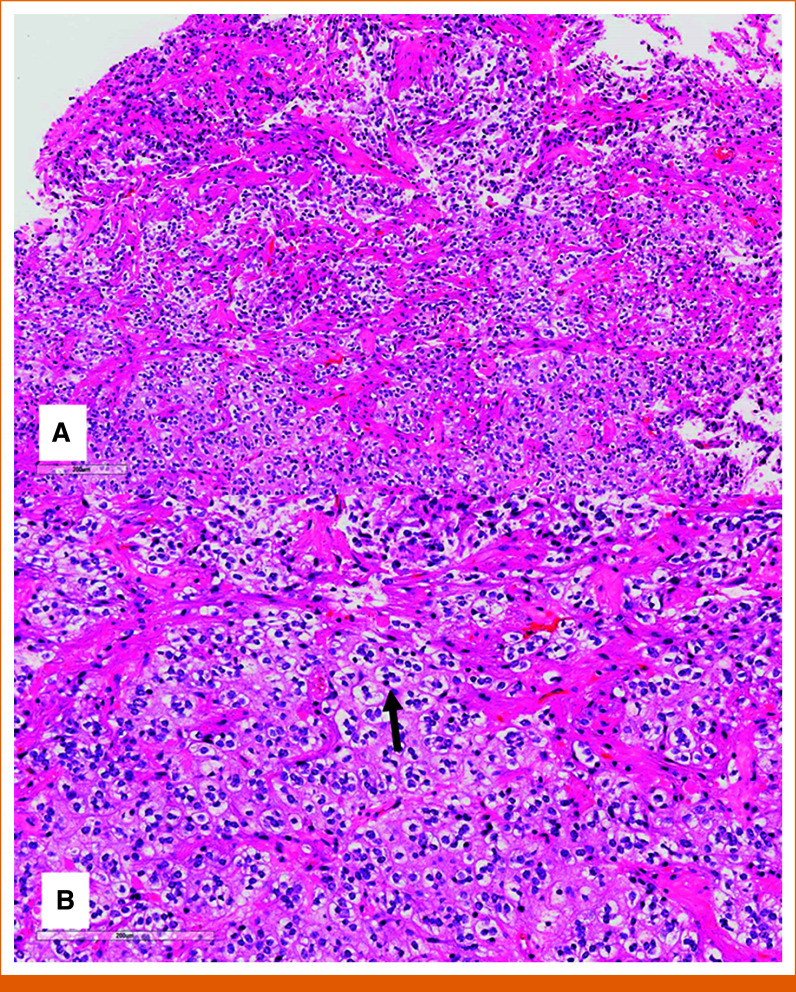
Hematoxylin and eosin stained histology images showing (A) a biphasic morphology with compact fibrillary areas alternating with looser regions. (B) Many of the tumor cells in the looser areas have round, monomorphous nuclei, perinuclear clearing, and there are examples of a pennies-on-a-plate morphology (black arrow).

Pathology from her first surgery was consistent with PA, WHO grade 1. The microscopic sections showed the classic biphasic morphology of PA with areas of densely fibrillary background and elongated glial tumor nuclei alternating with areas of lesser cellularity with many tumor cells being round with examples of a pennies-on-a-plate pattern (Fig 2). On immunohistochemical staining, the fibrillary cytoplasmic processes immunolabeled with GFAP, the tumor cells were immunonegative for *mIDH1 (R132H)* and *BRAF V600E*, ATRX expression was retained, p53 labeling was variable, and the Ki67 proliferative index was approximately 10%. Molecular testing via Illumina TruSight RNA Pan-Cancer next-generation sequencing (NGS) did not reveal any *BRAF* fusions, but did show an oncogenic *KRAS* p.Q61R single-nucleotide variant.

Her case was discussed at an institutional multidisciplinary tumor board and a national AYA molecular tumor board (MTB; Canadian AYA Neuro-Oncology Network). The consensus was that a trial of MEK inhibition using trametinib was reasonable given the otherwise large treatment field for radiation therapy. Through a compassionate access drug program, she was started on trametinib at an initial dose of 1 mg once per day, which was then increased to 1.5 mg once daily. She developed facial and truncal acneiform rash as a dermatological adverse effect of the drug. This was addressed with oral clindamycin and HydroVal 0.2% cream and is now well under control. Because of grade 3 aceniform rash, further escalation to the standard adult dose of 2 mg once daily was deferred after shared decision making to ensure better tolerability. Within 2 weeks of treatment, she had symptomatic relief of her headaches and improvement in her vision. Serial imaging was done, and her MRI after 6 months of therapy demonstrated an objective radiographic response (Fig 1).

## Patient Publication Consent

The authors declare that informed consent to publish information and images was obtained from the patient.

## Discussion

To the best of our knowledge, this is the first reported case of an oncogenic *KRAS* p.Q61R mutation in a noncerebellar PA with an objective response to trametinib. Advances in molecular diagnostics have increased our understanding of PA and have informed the treatment approach for this tumor type. MAPK pathway alterations are implicated in the majority of PA with the most common finding being *KIAA1549::BRAF* fusion (*BK* fusion).^3^ Other genetic alterations include other *BRAF* mutations or fusions, *NF1* mutations, *FGFR1* mutations or fusions, and NTRK fusions.^6^ Rarely, *KRAS* mutations, *RAF1* fusions, *ROS1* fusions, and other alterations of *MET* and *RET* are identified.^1^ There is an association between certain genetic alterations and the location of PA; for example, *BRAF* fusions are more frequent in the posterior fossa than in supratentorial lesions.^3^ This has been demonstrated in pediatric low-grade gliomas (pLGG), and *BK* fusion was shown to be an independent favorable prognostic factor.^7^ There is also a correlation between age and *BRAF* fusions as they seem to be less frequent in adults.^8^

The Kirsten rat sarcoma viral oncogene homolog (KRAS) protein is an essential intracellular signal transducer involved in several cellular pathways. Oncogenic KRAS therefore disrupts normal biological processes.^9^ In particular, it leads to increased proliferation rates of cells harboring the mutation through the constitutive activation of the MAPK pathway.^10^ *KRAS* mutations have been detected in numerous tumors and are frequent in pancreatic, colorectal, and lung carcinomas, but are very rare in PA.^9^ Only two cases out of a population-based cohort of 1,000 pLGG had a *KRAS* mutation, suggesting that it is an uncommon alteration.^3^ The *KRAS* p.Q61R mutation, located in the catalytic G-domain of the protein, is known to be oncogenic and is found in pancreatic, colorectal, thyroid, and lung cancers.^11^ Table 1 summarizes a few reported cases of PA with *KRAS* mutations.^12-16^

**TABLE 1.** Review of Reported KRAS Mutations in Pilocytic Astrocytoma

Citation	Age, Years	Sex	Tumor Location	KRAS Mutation	Other Alterations
Cin et al^12^	11	—	Cerebellum	*KRAS E63K*	None
	1	—	Third ventricle	*KRAS G12A*	None
Jones et al^13^	40	Female	Fourth ventricle	*KRAS R73M*, *KRAS E63K*	None
	22	Female	Cerebellum	*KRAS L19F*, *KRAS Q22K*	None
Theeler et al^14^	46	Female	Thoracic	*KRAS G12S*	*KIAA1549::BRAF* fusion
Reinhardt et al^15^	—	Female	Spinal	*KRAS Q61H*	None
	—	Female	Posterior fossa	*KRAS V14A*	None
Chau et al^16^	24	—	Tectum	*KRAS E63K*	None

PA has favorable clinical outcomes with 10-year-survival rates of up to 95%.^1^ The mainstay of treatment is gross total resection, which is not always feasible. For those with residual tumor, treatment recommendations in the 2023 National Comprehensive Cancer Network Guidelines for CNS Cancers include observation, consideration of radiotherapy for those who develop significant tumor growth and neurologic symptoms, or use of BRAF and MEK inhibitors in the presence of a *BRAF V600E* mutation.^17^ Prospective studies evaluating radiotherapy and chemotherapy in the adjuvant and recurrent setting among adult patients are lacking, and the effectiveness of pediatric chemotherapy regimens in adults is unknown.^18^ Moreover, there is risk of gonadal toxicity and higher risk of vincristine neurotoxicity in AYA and older adults when using the standard pediatric chemotherapy regimen of vincristine and carboplatin. As such, multidisciplinary tumor board discussion is crucial to guide treatment.

Two KRAS inhibitors, sotorasib and adagrasib, have been approved recently for use in *KRAS G12C*-mutant lung cancer.^19,20^ The role of KRAS inhibitors in targeted glioma therapy is unclear. Unfortunately, there is currently no effective treatment for primary brain tumors that targets *KRAS* and has demonstrated good intracranial activity. *KRAS* mutations in tumors may respond to MEK inhibitors such as trametinib, cometinib, or binimetinib. As MEK is a downstream effector of the MAPK pathway, MEK inhibitors have potential efficacy against tumors with *RAS* mutations and class I or II *BRAF* mutations. Monotherapy with a MEK inhibitor showed promising outcomes in pLGG with *KIAA1549::BRAF* fusion or *NF1* loss of function.^5^ Meanwhile, dual therapy with dabrafenib and trametinib was found to be more effective and safe than standard chemotherapy with carboplatin plus vincristine as first-line therapy in pediatric patients with *BRAF V600* mutant low-grade gliomas.^4^

Here, we describe the case of an AYA patient diagnosed with a *KRAS*-mutant PA in an unfavorable location treated with up-front targeted therapy using downstream signaling pathway blockade which had a positive outcome. MEK inhibition in a *KRAS*-mutant PA is a promising therapeutic strategy to postpone radiotherapy and thus avoid the risk of long-term neurocognitive impairment in a patient likely to have good long-term survival with appropriate treatment. The duration of MEK inhibition and durability of the treatment response in this patient is unknown, and longer follow-up is required.

The optimal approach to *KRAS*-mutant targeted therapy is unclear, and as demonstrated in this case, downstream inhibition may be considered. Additional data regarding the efficacy and outcomes of patients treated with molecularly directed therapies are needed especially in the AYA population which has unique clinical, molecular, and treatment considerations.^21^ This case suggests that the use of molecular testing such as NGS panels to identify targetable genetic alterations and therefore direct the selection of treatment is an important step in the management of AYA patients.^22^ Furthermore, this case illustrates the utility and impact of an MTB discussion on the interpretation and correct matching of targeted therapy to a genetic alteration. Finally, this case reiterates the challenges and treatment gap in the care of neuro-tumor AYA patients that remain to be addressed.

In conclusion, we present a case of an AYA patient with a PA harboring an unusual *KRAS Q61R* mutation in which up-front targeted therapy using a MEK inhibitor led to an impressive radiographic response and clinical improvement. In patients with excellent long-term survival, there is a need to identify potential adjuvant therapies with much less toxic side effects than the conventional radiotherapy and chemotherapy strategies available. Up-front targeted therapy is a promising treatment strategy to avoid or postpone these risks without compromising outcomes. MTB discussions are valuable in treatment selection, particularly in the AYA group, which is regarded as an orphan group with unique host and disease biology and age-related challenges.
